# The unrevealed links: periodontal health, human milk composition, and infant gut microbiome dynamics

**DOI:** 10.1017/S1463423624000215

**Published:** 2024-11-14

**Authors:** Rana Badewy, Michael Glogauer, Kristin L. Connor, Michael Sgro, Jim Yuan Lai, Richard P. Bazinet, Howard C. Tenenbaum, Amir Azarpazhooh

**Affiliations:** 1 Faculty of Dentistry, University of Toronto, Toronto, ON, Canada; 2 Department of Dental Oncology, Princess Margaret Cancer Centre, Toronto, ON, Canada; 3 Department of Health Sciences, Carleton University, Ottawa, ON, Canada; 4 Department of Pediatrics, and Li Ka Shing Knowledge Institute, St. Michael’s Hospital, Unity Health Toronto, Toronto, ON, Canada.; 5 Department of Pediatrics, Division of Neonatology, University of Toronto, Toronto, ON, Canada; 6 Institute of Medical Sciences, Temerty Faculty of Medicine, University of Toronto, Toronto, ON, Canada; 7 Department of Nutritional Sciences, University of Toronto, Toronto, ON, Canada; 8 Department of Dentistry, Centre for Advanced Dental Research and Care, Mount Sinai Hospital, Toronto, ON, Canada

**Keywords:** Breast/human milk, breastfeeding, maternal health/infections, periodontal diseases, periodontitis

## Abstract

**Aim:**

This review aims to identify the mechanistic relationships related to periodontal diseases and its possible association with changes in human milk composition and the composition and function of infants’ gut microbiome.

**Background:**

Maternal health conditions, especially inflammatory, are associated with altered human milk composition. It is *not* known whether maternal oral inflammatory diseases, including periodontal diseases, deleteriously affect human milk composition.

**Methods:**

A narrative review was conducted according to SANRA, the Scale for the Assessment of Narrative Review Articles, guidelines. PubMed, Google Scholar, and Cochrane database of systematic reviews were searched from September 2019 up to December 2023 using keywords such as breast/human milk, maternal health/infections, and periodontal diseases. Reference lists of relevant articles were also screened. Our primary outcome of interest was human milk composition (i.e., any changes in macronutrients, immunological components, etc.). Secondary outcomes included changes in human milk microbiome and subsequent changes in the infant gut microbiome. Outcomes were synthesized using a narrative approach where the existing evidence and current literature were summarized. No risk of bias assessment of the studies was performed in this review.

**Findings:**

The search yielded no studies investigating the relationship between periodontal diseases in nursing mothers and changes in human milk composition. However, a dose–response relationship exists between the severity of periodontal diseases and the risk of adverse pregnancy outcomes such as preterm birth. Mastitis and diabetes affected milk lipids. Immunoglobulin A (sIgA) was increased in mastitis, whereas reduced concentrations were reported in diabetes. Potential biological pathways through which periodontal diseases can negatively affect human milk composition include the systemic dissemination of inflammatory cytokines like IL-6, PGE2, and tumor necrosis factor (TNF)-β that can be up-regulated by bacterial by-products. This biological plausibility needs to be investigated, given the potentially negative impact on the quality of human milk that could be caused by periodontal inflammation.

## Introduction

Human milk is critical for optimal newborn growth and development (Andreas *et al*., 2015). Human milk contains bioactive substances including polyunsaturated fatty acids (PUFAs), immune cells, hormones, growth, and immunological factors like cytokines and immunoglobulins (Andreas *et al*., 2015). Infants nursed with human milk during their first months of life are less vulnerable to infections, especially those originating in the gastrointestinal and respiratory tracts (Pullan *et al*., 1980) than those who received formula. However, the mechanisms involved in the transmission of immunity through human milk are still not fully understood. In addition, human milk contains its own microbiome and human milk oligosaccharides that likely shape the infants’ gut microbiome, affecting infant health outcomes both early and later in life (Fitzstevens *et al*., 2017). There is emerging evidence that supports the role of the human milk microbiome in regulating microbial colonization of the infant gut with health-promoting microbes (Fernández *et al*., 2013), decreasing the likelihood of allergies (Fernández *et al*., 2013), irritable bowel syndrome (Fernández *et al*., 2013), elevated fat mass (Hunt *et al*., 2011, Sanz, 2011), and other diseases (Sanz, 2011) in the child. However, the specific human milk components that promote and maintain optimal growth and health, and the mechanisms through which these are transmitted to the infant, are incompletely understood.

While nursing, some mothers (referred to as birthing parents thereafter for an inclusive language) may develop infections that could contribute to the overall systemic inflammation throughout the body (Offenbacher *et al*., 1996, Schieve *et al*., 1994). Inflammation and infection in the oral cavity and urinary tract in women (referred to as individuals/persons thereafter for an inclusive language) during pregnancy have been associated with adverse pregnancy outcomes such as preeclampsia, preterm birth, and low birth weight (Kass, 1962, Schieve *et al*., 1994, Baskaradoss *et al*., 2012, Ide and Papapanou, 2013), suggesting that infections during pregnancy have effects beyond the local niche. This consideration derived studies to further investigate the impact of maternal infections in the postpartum period on the composition of human milk.

Maternal health conditions such as diabetes and mastitis have been shown to be associated with altered human milk composition (Hunt *et al*., 2013, Groer *et al*., 2004, Morceli *et al*., 2011, Smilowitz *et al*., 2013, Peila *et al*., 2020), although as regard to the latter this is easier to explain. These alterations in human milk can potentially affect the transmission of immune factors to the nursing infant and have serious implications for the infant’s health (Zárate *et al*., 2017, Gurven *et al*., 2016). For example, infants exposed to elevated levels of PUFAs in the first months of their life are at risk of childhood obesity (Zárate *et al*., 2017). Furthermore, elevated counts of cytokines have been associated in the literature with cardiovascular diseases, metabolic syndromes, and type 2 diabetes (Gurven *et al*., 2016).

During pregnancy, oral inflammatory conditions such as gingivitis and periodontitis affect 30–100% and 20–50% of pregnant persons, respectively, and both conditions affect 15% of individuals of childbearing aged (Patil *et al*., 2012, Laine, 2002). Despite that the relationships between periodontal diseases and adverse pregnancy outcomes are well documented (Daalderop *et al*., 2018, Iheozor-Ejiofor *et al*., 2017), it is not known whether periodontal diseases affect human milk composition negatively. The extent to which human milk composition is influenced by periodontal diseases needs to be investigated, a key gap in understanding the link between suboptimal oral health and infant outcomes.

Here, we aimed to identify the main immunological components in human milk that are essential for the infants’ growth and development and the impact of maternal systemic diseases on human milk composition. We also aimed to synthesize the evidence on the mechanistic relationships and mechanisms between periodontal diseases and human milk composition. We also aimed to identify and discuss the mechanisms through which periodontal diseases in nursing individuals may influence both the composition and quality of human milk and consequently, the infant health. The fact that periodontal inflammation could negatively impact the composition of human milk (i.e., harming nutritional and immunological contents (Groer *et al*., 2004, Hunt *et al*., 2013)) is biologically plausible. Recognizing this gap of knowledge regarding the association between periodontal diseases in birthing parents and their human milk composition, we conducted the following comprehensive review.

## Methods

This review of the literature was conducted from September 2019 to December 2023 by searching PubMed, Google Scholar, Cochrane database of systematic reviews, and screening reference lists of relevant articles to investigate the potential relationship between periodontal diseases in birthing parents and human milk composition. This review was prepared considering SANRA, the Scale for the Assessment of Narrative Review Articles, checklist as a guideline (Baethge *et al*., 2019). In particular, this review aims to answer the following questions:

1. What are the main immunological components in human milk and how are these elements essential for the infants’ growth and development?
2. What is the impact of maternal systemic diseases on human milk composition?
3. What are the mechanistic relationships and mechanisms described in the literature related to periodontal diseases and its possible association with changes in human milk composition?
4. How maternal health plays a major role in the development of the infants’ intestinal microbiome and the potential impact of periodontal disease in birthing parents on the composition and function of their infants’ gut microbiomes.
5. What are the implications for future research, practice, and policy development related to the dental and medical field.

The keywords used in the search strategy included breast milk, human milk, breastfeeding, maternal health/infections, periodontal diseases, gingivitis, periodontitis, oral inflammation, and maternal oral health. No study design restrictions were applied; however, the search was limited to studies published only in English language.

Our search strategy was guided by the components of the PECO model (Population, Exposure, Comparison, and Outcome) outlined below:

- Population: Pregnant/breastfeeding individuals aged 18 years or older
- Exposure: Maternal infections/diseases/periodontal diseases (gingivitis and periodontitis)
- Comparison: Healthy individuals/individuals without infections/periodontal diseases
- Outcome: 1. Primary outcome included any changes in human milk composition (nutritional or immunological components, etc.) 2. Secondary outcome included changes in human milk microbiome and subsequent changes in infant gut microbiome.

Outcomes were synthesized using a narrative approach where the existing evidence and current literature were summarized. No risk of bias assessment of the studies was performed in this review.

## Results

### Human breast milk’s main immunological components and infant outcomes

Human milk contains many immunological and nutritional components that are paramount for the infants’ growth and development and immune responses of newborns during the challenging period of immunological adaptation to extra-uterine life. Human milk contains long-chain PUFAs, immunoglobulins, cytokines, immune cells, and other factors that help reduce a newborn’s susceptibility to infections and also facilitate its growth and development (Garofalo, 2010, Gilmore *et al*., 1994, Andreas *et al*., 2015) (Table 1). The difference between breast-fed and formula-fed infants in terms of infants’ susceptibility to infections has been discussed in a variety of studies (Feachem and Koblinsky, 1984). A meta-analysis showed that the incidence of diarrhea in breastfed infants is four- to five-fold lower during the first six months of life compared to infants fed with formula (Feachem and Koblinsky, 1984, Morrow and Rangel, 2004). Respiratory infections and death due to infections were also less common in breastfed infants when compared to formula-fed infants (Feachem and Koblinsky, 1984). Accordingly, breastfeeding is conceivably a powerful preventive measure against morbidity and mortality caused by infectious diseases in young children (Morrow and Rangel, 2004, Clavano, 1982).

**Table 1.** Summary of breast milk main nutritional and immunological components

Breast milk components	Types and concentrations	Suggested role in infants’ growth
Immune cells (Andreas *et al*., 2015)	Macrophages (55–60%), neutrophils (30–40%), and lymphocytes T and B (5–10%) are found in human milk	• Help the infant develop a functional immune system through childhood. • The infant’s T-cell activity is stimulated by the help of macrophages which provide adequate protection against pathogens.
Cytokines (Murphy *et al*., 2018, Garofalo, 2010)	Interleukin-1 beta (IL-1b), IL-6, tumor necrosis factor-alpha (TNF-a), interferon-g (IFN-g), IL-10, transforming growth factor-beta (TGF-b)	• IL-1b pro-inflammatory cytokine is involved in the host defense response to injury and recruitment of neutrophils to the mammary gland. • IL-6 pro-inflammatory cytokine has an essential role in the development of the infant’s mucosal immunity. • Tumor necrosis factor-alpha (TNF-a), an important regulator of immune cells, is involved in the chemotactic activity of neutrophils and stimulates human milk’s IL-6 production. • TGF-b has been associated along with IL-6 and IL-10 in the development and differentiation of immunoglobulin A (IgA) producing cells in the mammary gland. It has also been linked with the maturation of the intestinal immune system as well as preventing allergic reactions. • Interferon-g (IFN-g), a cytokine with immuno-stimulatory and immuno-modulatory effects, is also capable of inhibiting virus replication.
Colony-stimulating factors (Gilmore *et al*., 1994)	Granulocyte-colony-stimulating factor (G-CSF), macrophage-colony-stimulating factor (M-CSF), and granulocyte-macrophage-colony-stimulating factor (GM-CSF)	Play a significant role in fetal intestinal development as well as having a beneficial effect on the proliferation, differentiation, and survival of neutrophils.
Immunoglobulins (Andreas *et al*., 2015)	Human breast milk is rich in immunoglobulins (IgA, IgM, and IgG), the most prominent of which is the secretory immunoglobulin A (sIgA)	Protect the infant at the mucosal level by interrupting virus replication, activating the complement system, and promoting phagocytosis.
Lactoferrin (Andreas *et al*., 2015)	A protein with antioxidant, bacteriostatic, and bactericidal properties	• Regulating immune and inflammatory responses. • Efficient in killing many different pathogens without inducing inflammation.
Oligosaccharides (Andreas *et al*., 2015)	The third largest component in breast milk, accounting for 12.9 g/L on average in mature milk and 20.9 g/L at four days postpartum	They act as prebiotics stimulating the proliferation of beneficial bacteria such as *Bifidobacterium infantis* in the intestines of infants essential for the enhancement of the intestine defense mechanisms against various pathogens.
Lipids (Andreas *et al*., 2015)	One of the major nutritional components of human milk is considered the major source of energy in human milk representing 40–55% of its overall energy.	Essential for central nervous system myelination due to the presence of Sphingomyelins which exist in the membrane of the milk fat globule
Polyunsaturated fatty acids (PUFAs) (Andreas *et al*., 2015)	Human milk is particularly rich in EFAs, GLA, DGLA, AA, EPA, and DHA.	DHA, EPA, and AA are the major LCPUFAs found in the brain and retina. Throughout childhood, neurological and immunological developments rely on the action of these fatty acids. It has been demonstrated that they also play a major role in the brain’s growth spurt occurring from the last trimester of pregnancy to age of 2.

EFAs, essential fatty acids; GLA, gamma-linolenic acid; DGLA, dihomo-gamma-linolenic acid; AA, arachidonic Acid; EPA, eicosapentaenoic acid; DHA, docosahexaenoic acid; LCPUFAs, long-chain polyunsaturated fatty acids.

### Maternal systemic diseases influence human milk composition and quality

#### Diabetes

Diabetes is a metabolic disease that induces immunological and biochemical changes in human milk (Morceli *et al*., 2011). For example, as compared to non-diabetic persons, lower levels of complement component 3 protein (acting as opsonins, which aid in in phagocytosis performed by cells of the immune system) was noted in the colostrum of diabetic persons. In addition, reduced levels of secretory immunoglobulin A (sIgA) and IgG were reported in the human milk of diabetic birthing parents compared to their non-diabetic counterparts (Smilowitz *et al*., 2013), whereas glucose and lipase enzymes were represented in higher amounts (Morceli *et al*., 2011). It has been hypothesized that hyperglycemia can affect B-lymphocytes in the serum of diabetic birthing parents, thereby reducing the antibody levels (Smilowitz *et al*., 2013). Another explanation is that prolactin plays a significant role in humoral immunity by binding to prolactin receptors on B-lymphocytes to stimulate the synthesis of immunoglobulins, and since the concentrations of prolactin are reduced in birthing parents with diabetes, this can impair the secretion of immunoglobulins (Smilowitz *et al*., 2013, Russell *et al*., 1985). These findings are consistent with other studies reporting significant 63.6% reduction in the concentration of sIgA in the colostrum of individuals with gestational diabetes mellitus (GDM), compared to those without GDM (Morceli *et al*., 2011, Smilowitz *et al*., 2013, Peila *et al*., 2020).

Given that diabetes is a condition of low-grade systemic inflammation and disruption in the immune system regulation promoting inflammatory responses, changes in the concentrations of cytokines, chemokines, and growth factors in the colostrum of postpartum individuals who had GDM have also been investigated in recent studies (Avellar *et al*., 2022). Higher concentration of IL-10, interferon-g (IFN-g), IL-15, and IL-6 were observed in the GDM birthing parents’ colostrum, compared to healthy birthing parents (Avellar *et al*., 2022). With respect to growth factors’ concentration, a statistically significant difference was observed in the level of GM-CSF growth factor between healthy and GDM birthing parents, where lower levels were found in the GDM birthing parents’ colostrum (Avellar *et al*., 2022). Growth factors in human milk are known to have a significant and pivotal role in the maturation of the infants’ intestinal mucosa (Murphy *et al*., 2018). Therefore, new studies should focus on how these changes can impact the infant’s health throughout their life. Further, a recent study (Choi *et al*., 2022) showed alterations in bioactive components in human milk (e.g., insulin, glucose, and C-reactive protein (CRP)) of birthing parents with GDM, where higher levels of CRP and lower levels of insulin and glucose were observed in the GDM birthing parents’ milk at both 1 and 3 months postpartum, compared to birthing parents without GDM.

Macronutrients including fats, proteins, and carbohydrates are also key components of the human milk and are shown to be altered by the metabolic state of the mother (Zhong *et al*., 2022, Korkut *et al*., 2022). For example, human milk from diabetic birthing parents has low fat content compared to non-diabetic birthing parents (Bitman *et al*., 1989). With respect to the fatty acid content, there was a four-fold increase of lipoprotein lipase and increase in the free fatty acids counts (Bitman *et al*., 1989). Another notable change in the fatty acids composition in the milk of diabetic birthing parents was a decrease in medium-chain fatty acids and an increase in the PUFAs compared to milk from birthing parents without diabetes, which could be the results of improper fatty acid synthesis in the mammary gland and an increase in the hydrocarbon chain elongation, respectively (Bitman *et al*., 1989). These alterations in lipid metabolism within the mammary glands might be related to changes caused by diabetes, since insulin is known to be involved in fatty acids synthesis through regulating the desaturation and elongation enzymes (Azulay Chertok *et al*., 2017). Infants exposure to high levels of fatty acids may contribute to development of obesity (Zárate *et al*., 2017), atherosclerosis, and diabetes later in life and may influence the neonatal neurodevelopment (Keim *et al*., 2012) in part through the prothrombotic and pro-inflammatory actions of PUFAs and their major role in controlling adipogenesis (Keim *et al*., 2012, Zárate *et al*., 2017). Another study (Korkut *et al*., 2022) that investigated the changes in the macronutrient content in human milk reported an increase in the carbohydrate content in the colostrum of GDM birthing parents, compared to their non-GDM counterparts, whereas no differences were observed in the fat and protein content between the study groups.

Some studies have shown interest in investigating the impact of GDM on the concentrations of metabolic hormones (e.g., adiponectin, leptin, ghrelin, insulin, apelin, etc.) in human milk. A recent systematic review of 12 studies (Suwaydi *et al*., 2022) showed that although evidence in this area is scarce, the synthesis suggested reduced levels of adiponectin, ghrelin, and irisin in human milk of individuals with GDM, especially during the early stages of lactation. Another recent study (Dou *et al*., 2023) focused on investigating the difference in human milk oligosaccharides between GDM and non-GDM birthing parents and showed that the total level of the identified 14 oligosaccharides was higher in the colostrum of GDM birthing parents compared to healthy ones. Further, with respect to changes in the level of individual oligosaccharides, lacto-N-neotetraose (LNnT) was significantly higher in GDM birthing parents in the colostrum, transitional, and mature milk.

#### Mastitis

Mastitis is a common inflammatory disease in lactating persons, affecting between 10 and 27% of individuals (Ingman *et al*., 2014) and is generally related to infection. Since bacteria plays a role in breast inflammation caused by mastitis, recent studies focused on identifying the bacterial factors of mastitis and evaluate their effects on the physical (i.e., sugar, protein, and fat) and chemical (i.e., pH, density, and freezing temperature) composition of human milk (Shuyang and Qiang, 2021). Birthing parents experiencing mastitis caused by Staphylococcus aureus bacteria exhibited lower sugar levels in their milk, while those with coagulase-negative staphylococci had reduced protein content (Shuyang and Qiang, 2021). This suggests that Staphylococcus aureus might reduce milk sugar through consumption, and coagulase-negative staphylococci may also affect milk protein (Shuyang and Qiang, 2021). In another study, milk produced from symptomatic breasts was shown to have greater lipolysis, generating elevated counts of free fatty acids (Hunt *et al*., 2011, Say *et al*., 2016). This was possibly as a result of the action of lipase enzymes produced by infiltrating leukocytes in response to inflammation. Since free fatty acids and monoglycerides of human milk have antibacterial properties, an increase in their numbers might reflect the host’s response to bacteria-induced disease (Hunt *et al*., 2013). In the presence of mastitis, levels of interleukin (IL)-6, lactoferrin, sIgA, and milk fat globule size were also shown to be higher in comparison to milk from healthy individuals, and the difference in size was larger if accompanied by systemic symptoms like fever (Mizuno *et al*., 2012). However, it has been proposed that the elevation of certain milk components (e.g., the pro-inflammatory cytokines and immunoglobulins) caused by mastitis might also protect the nursing infant from developing infectious illnesses such as Group B Streptococcus neonatal infection (Buescher and Hair, 2001).

#### Allergic diseases

Inflammation associated with allergic diseases (e.g., atopic eczema, asthma, and allergic rhinitis) is known to affect the production of inflammatory mediators in the blood (Galli *et al*., 2008); however, it is unclear whether these diseases also affect their production or levels in human milk. Concentrations of cytokines that are related to allergic reactions and the production of sIgA (e.g., IL-4, -5, -10, and -13) are higher in the human milk of allergic birthing parents compared to their non-allergic counterparts (Böttcher *et al*., 2000). The data on PUFA concentrations in human milk with maternal atopy are inconsistent. One study observed reduced levels of long-chain n-3 PUFAs in human milk of atopic birthing parents compared to non-atopic birthing parents, despite that birthing parents with atopy in this study consumed high amounts of fish rich in long-chain PUFAs (Johansson *et al*., 2011). The decrease in human milk PUFAs was suggested to be due to the consumption or exposure to environmental or food allergens, leading to inflammation and overproduction of prostaglandins and leukotrienes that upregulate inflammation, thereby altering the function of mammary gland cells and ultimately the content of human milk (Johansson *et al*., 2011). However, a second study did not find any differences in human milk PUFA concentrations between birthing parents with and without atopy (Lauritzen *et al*., 2006).

#### Obesity and body composition

A number of studies investigated the impact of maternal obesity and body mass index (BMI) on the macronutrients (Daniel *et al*., 2021, Leghi *et al*., 2020), fatty acids (de la Garza Puentes *et al*, 2019), and bioactive components (e.g., insulin, leptin, CRP, and osteopontin) (Sims *et al*., 2020, Ruan *et al*., 2022, Zhu *et al*., 2022) on human milk. In comparison to non-obese/normal weight birthing parents, human milk from obese birthing parents (BMI ≥25 kg/m^2^) (Ellsworth *et al*., 2020) was found to have higher fat and lactose concentrations; however, there were no differences in the protein concentrations (Leghi *et al*., 2020, Daniel *et al*., 2021, Froń and Orczyk-Pawiłowicz, 2023). With respect to human milk fatty acids concentration, human milk from obese birthing parents had elevated levels of saturated fatty acids and lower levels of PUFAs (α-linolenic acid (ALA) and docosahexaenoic acid (DHA)) and monounsaturated fatty acids compared to human milk from birthing parents with normal weight (de la Garza Puentes *et al*., 2019, Tekin-Guler *et al*., 2023). However, studies show conflicting results with respect to the PUFAs concentrations in the milk of obese birthing parents. A recent study (Ellsworth *et al*., 2020) showed higher levels of dihomo-gamma-linolenic acid (DGLA) and arsenic omega-6 PUFAs in the milk of obese birthing parents compared to birthing parents with normal weight. A review paper (Bardanzellu *et al*., 2020) found changes in eight specific human milk metabolites, including nucleotide derivatives, 5-methylthioadenosine, sugar alcohols, acylcarnitine and amino acids, polyamines, mono- and oligosaccharides, and lipids, in overweight or obese birthing parents compared to lean birthing parents. Higher levels of insulin (Ramiro-Cortijo *et al*., 2023, Schneider-Worthington *et al*., 2021), leptin (Andreas *et al*., 2014, Schneider-Worthington *et al*., 2021, De Luca *et al*., 2016), CRP, and lower levels of oligosaccharides (Saben *et al*., 2021, Froń and Orczyk-Pawiłowicz, 2023) have also been reported in the human milk of obese birthing parents compared to normal-weight birthing parents (Sims *et al*., 2020). Further, pre-pregnancy BMI of birthing parents exhibited a positive correlation with osteopontin levels early postpartum (Zhu *et al*., 2022), and a positive correlation was also found between osteopontin and maternal body composition factors, including body weight, bone mineral content, skeletal muscle mass, body fat, and visceral fat area (Ruan *et al*., 2022).

Given the significant role of human milk microbiome in shaping the infants’ gut microbiome which can have implications for infants’ health and development, recent studies focused on investigating the links between maternal weight status and the human milk microbiome composition. A recent scoping review of 20 longitudinal and cross-sectional studies showed that some studies reported that overweight or obese individuals displayed higher levels of Staphylococcus genus, reduced abundance of Bifidobacterium, and lower alpha diversity (within-sample diversity) (Daiy *et al*., 2022). It has been suggested that the above-mentioned changes in the human milk composition of obese birthing parents can put their infants’ at high risk of childhood obesity and complicated health outcomes (Leghi *et al*., 2020, Lecoutre *et al*., 2023, Bardanzellu *et al*., 2020). However, more robust longitudinal studies are needed to further evaluate the impact of maternal obesity on the infants’ health outcomes.

#### Acute kidney injuries

Although there are limited data, some studies show human milk composition can be negatively affected by acute kidney injuries, which may be a consequence of systemic inflammation, changes in pH, and accumulation of metabolic wastes associated with the disease (Chruscicki *et al*., 2017). A 2017 case report (Chruscicki *et al*., 2017) found a decrease in the amino acid concentrations (alanine, glutamine, glutamate, glycine, and valine) in the human milk of a mother with acute kidney injuries and an increase in the levels of creatinine and other catabolites compared to milk from healthy birthing parents, whereas the levels of macronutrients, including lipids and carbohydrates, were similar to their levels in health birthing parents. In early acute kidney injuries, there is increased protein catabolism which may be the reason for decreased amino acid levels in the human milk (Chruscicki *et al*., 2017). More studies are required to examine the potential relationship between kidney injury and human milk composition.

#### Celiac disease

Celiac disease (CD) is a serious autoimmune disease where the intestine of affected individuals is highly sensitive to food containing gluten (Olivares *et al*., 2015, Roca *et al*., 2018). Studies reported lower levels of immunological factors in human milk including the transforming growth factor (TGF)-β1 and sIgA in the milk of birthing parents with CD, compared to healthy birthing parents (Olivares *et al*., 2015). It has been proposed that this reduction in the milk’s protective factors can put the infant at risk of developing CD later in life (Olivares *et al*., 2015). On the other hand, human milk from birthing parents with CD who followed a gluten-free diet showed no difference in the levels of anti-gliadin antibodies (including IgA and IgG) from the milk of healthy birthing parents (Roca *et al*., 2018). A recent study investigated human milk microbiome composition in birthing parents with CD on a gluten-free diet and found in their human milk elevated levels of three bacterial strains, including one (Rothia mucilaginosa) that has been previously associated with autoimmune conditions, compared to healthy birthing parents (Olshan *et al*., 2021).

#### Preeclampsia

Preeclampsia is a systemic inflammatory diseases and a common pregnancy complication affecting 3–5% of pregnancies (Peila *et al*., 2022). Studies investigating the impact of preeclampsia on human milk composition are scarce; however, a recent review of 15 articles found alterations in the composition of human milk of preeclamptic birthing parents (Peila *et al*., 2022). Preeclamptic birthing parents had lower concentrations of cytokines (IL-6 and IL-8), elevated levels of adiponectin and DHA, and low vitamin A and E levels in their human milk compared to their healthy counterparts (Peila *et al*., 2022). However, some studies showed no differences in the macronutrient content (Beser *et al*., 2022) and activin A levels (a human milk neurobiomarker essential for central nervous system development) (Coscia *et al*., 2023) of human milk between birthing parents with and without preeclampsia.

#### Gestational hypertension

Gestational hypertension is another condition that has been recently mentioned in the literature to affect human milk lactogenesis and percentage composition (Sokołowska *et al*., 2023). Substantial variations were observed in the human milk composition of postpartum birthing parents with gestational hypertension when compared to healthy, normotensive counterparts (Sokołowska *et al*., 2023). Human milk from birthing parents with gestational hypertension exhibited elevated levels of fat, carbohydrates, and energy compared to healthy ones (Sokołowska *et al*., 2023). Future studies are needed to further investigate this association and assess the differences in growth rates of the infants.

#### Inflammatory bowel disease

Few recent studies show that birthing parents with inflammatory bowel disease had low abundance of *Bifidobacterium* and lower abundance of proteins involved in immune regulation, including thymic stromal lymphopoietin, IL-12 subunit beta, tumor necrosis factor (TNF)-beta, and C-C motif chemokine 20 in their human milk compared to birthing parents without inflammatory bowel disease (Sabino *et al*., 2023). These changes were shown to have a negative impact on the development of intestinal microbiota of infants.

#### Vaginal infections

Vaginal yeast infections during pregnancy can affect the immunological and antimicrobial properties of human milk (Nisaa *et al*., 2023). A recent study investigated the differences in microbiota profiles between birthing parents with and without vaginal yeast infections. Human milk from birthing parents with vaginal infections demonstrated increased alpha diversity at various taxonomic levels and elevated levels of Staphylococcus genus and Streptococcus infantis species, compared to birthing parents without infections (Nisaa *et al*., 2023).

### Pregnancy, postpartum period, and periodontal disease

#### Pregnancy effects on periodontal disease

**Figure 1.**
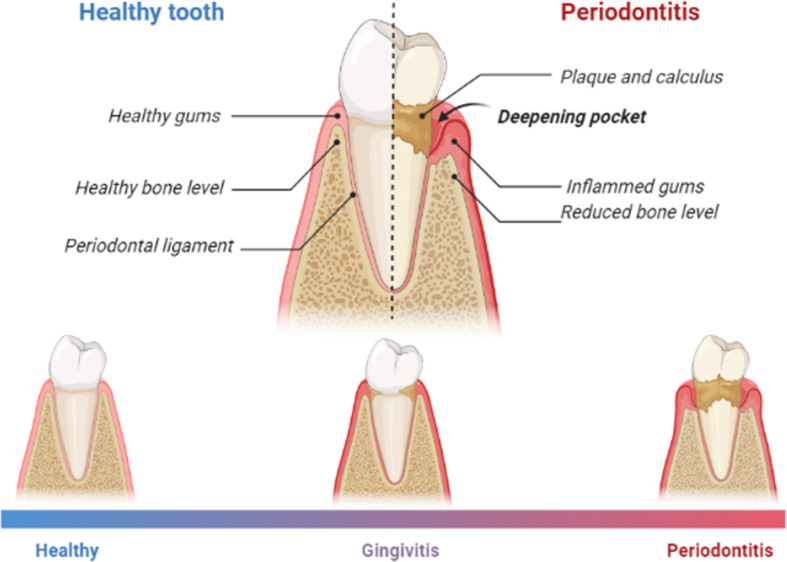
A diagram showing differences between healthy teeth and teeth with gingivitis and periodontitis. Gingivitis is a reversible inflammation confined to the gingival tissues. The teeth in the oral cavity are supported by a ligament known as the periodontal ligament which attached the teeth to the gingiva and surrounding alveolar bone. The space between the tooth and the gingival tissues is known as the gingival sulcus, and this is where most of the dental plaque accumulates, triggering an inflammatory response. What differentiates gingivitis from periodontitis is that the latter is an irreversible destruction to the periodontal supporting tissues, including the alveolar bone, periodontal ligament, and cementum, which consequently results in tooth loss (Kinane *et al*., 2017, Tatakis and Kumar, 2005). As sources of inflammation, periodontal diseases contribute to the overall inflammatory burden experienced by individuals (Khoury *et al*., 2020). Initially, the gingival response to the biofilm is presented in the form of redness, edema, and bleeding. Oral polymorphonuclear neutrophils are then recruited to the sites of inflammation. In a healthy periodontium, oral neutrophils are usually found at the junctional epithelium and base of the sulcus (Khoury *et al*., 2020) to protect the periodontal tissues by providing a barrier between the junctional epithelium and pathogens within the dental plaque. However, as bacterial counts increase, so does the oral neutrophils count which enhance their activity (Khoury *et al*., 2020). Any deviation from PMN’s normal production, recruitment, function, or activity can lead to damage in periodontal tissues, and consequently tooth loss. (Prepared using Biorender.com).

Periodontal diseases (gingivitis and periodontitis) are oral inflammatory conditions that are caused by bacterial plaque, and they result from a disruption in the normal interaction between the host and microflora in the oral cavity (Kinane *et al*., 2017, Khoury *et al*., 2020). Bacterial plaque is the oral biofilm that adheres to the tooth surface and gingiva and represents the main local etiologic factor for periodontal diseases (Figure 1) (Kinane *et al*., 2017, Tatakis and Kumar, 2005, Khoury *et al*., 2020).

Many host-related conditions can affect the severity of the periodontal disease among which are sex hormones that exacerbate the pathogenesis and progression of the disease (Tilling, 2014, Patil *et al*., 2012). Pregnancy is associated with dynamic changes in the immune, endocrine, and metabolic milieus of the pregnant person (Tilling, 2014), including changes in the levels of estrogen and progesterone, which reach a peak of 6 and 100 ng/ml (10–30 times higher than their levels during the menstrual cycle) by the end of the third trimester, respectively (Patil *et al*., 2012, Grodstein *et al.*, 1996). Some pregnancy-related changes occur in the oral cavity including gingivitis and periodontitis (Patil *et al*., 2012, Laine, 2002). The majority of studies reported that the pregnancy-related changes in the oral cavity are confined to gingival tissues (Tilakaratne *et al*., 2000, Tilling, 2014, Cohen *et al*., 1971) with studies reporting no changes in the periodontal attachment levels (Cohen *et al*., 1971). Therefore, pregnancy-related hormonal changes are believed to cause gingivitis during pregnancy; however, they do not likely affect periodontal tissues but may aggravate preexisting periodontal disease (Tilling, 2014). While the relationship between periodontal diseases and common adverse pregnancy outcomes is sufficiently established, this is still a need for more robust randomized clinical trials to confirm the association (Iheozor-Ejiofor *et al*., 2017).

While many mechanisms have been proposed to explain how pregnancy-related hormonal changes could increase the susceptibility to gingival inflammation, the reason is still unknown (Tilling, 2014). Proposed explanations include: 1. progesterone can increase the vascular permeability and decrease production of IL-6 by fibroblasts (Amar and Chung, 1994), 2. gingival tissue acts as a target for estrogen and progesterone due to the presence of their receptors in the gingival tissue (Offenbacher *et al*., 2001), 3. progesterone and estrogen have been associated with greater prostaglandin synthesis by macrophages and depressed antibody responses (Amar and Chung, 1994), 4. pregnancy-related hormonal changes are associated with greater polymorphonuclear neutrophils levels in the gingival sulcus and with an alteration of their function, thereby lowering the gingival resistance to bacterial invasion (Barriga *et al*., 1994), and 5. some bacterial species associated with gingival inflammation, including Bacteroides species and Prevotella intermedia are stimulated by pregnancy hormones (Barak *et al*., 2003).

#### Periodontal disease affects pregnancy outcomes

Recent evidence has suggested that there is a bidirectional relationship between periodontal diseases and pregnancy. Not only can pregnancy affect periodontal disease pathogenesis, but the opposite might also occur, and periodontal disease can affect pregnancy outcomes (Baskaradoss *et al*., 2012, Ide and Papapanou, 2013). As sources of inflammation, periodontal diseases contribute to the systemic inflammatory experienced by the patient (Offenbacher *et al*., 2001, Moore *et al*., 2004, Teshome and Yitayeh, 2016), and in the context of pregnancy, this heightened inflammation may have implications for inflammatory-associated processes, such as parturition (Daalderop *et al*., 2018). Indeed, the first study that reported a positive association between periodontal infection in individuals during pregnancy and preterm low birth weight showed that maternal periodontitis was associated with a seven-fold increased risk for the delivery of a preterm, low birth weight infant (Offenbacher *et al*., 1996). This finding was consistent with other future studies reporting an association between the severity of periodontal disease and increased odds of low birth weight delivery (Saddki *et al*., 2008, Khader *et al*., 2009). These associations, however, are still not entirely settled as evidenced by data shown in other studies reporting no significant association between oral health status and birth weight, except for birthing parents aged over 25 years, where maternal periodontitis was associated with low birth weight (Marin *et al*., 2005). This can be explained by the fact that age is considered as a predictor of periodontal diseases, so older individuals may have more severe periodontal diseases than younger individuals (Nazir, 2017).

**Figure 2.**
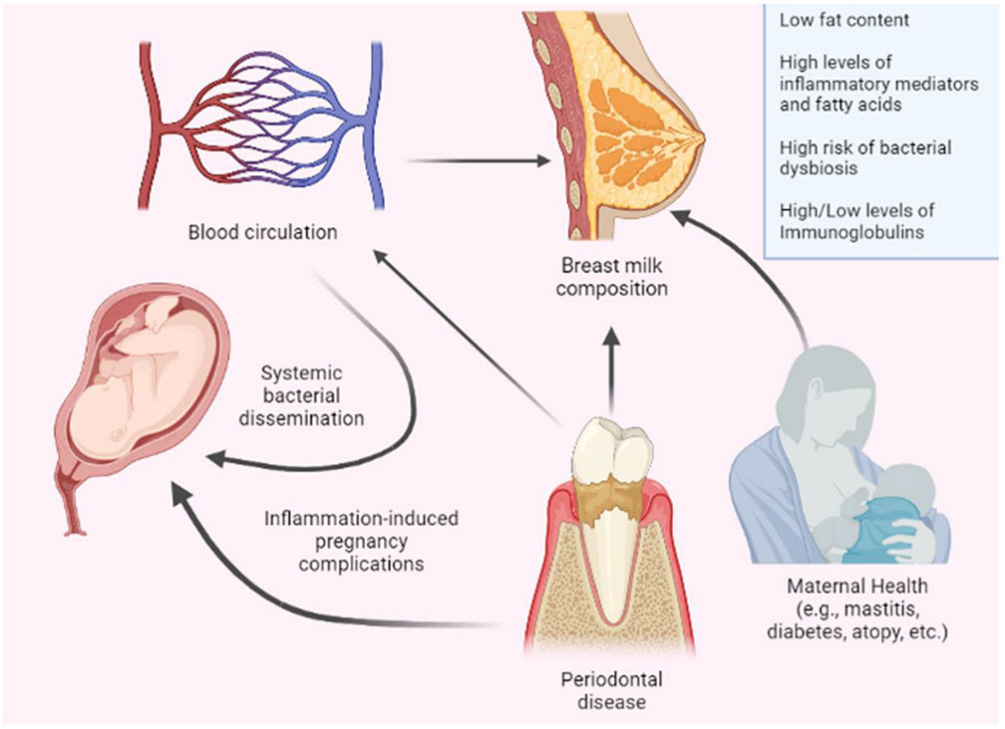
A simplified diagram showing the potential mechanism explaining the impact of periodontal disease on breast milk composition. Maternal diseases/infections (such as diabetes, mastitis, and atopy) can affect the composition of breast milk. Periodontal diseases have been reported to in the literature to be associated with adverse pregnancy outcomes, including preeclampsia, preterm birth, and low birth weight. Potential biological pathways through which periodontal diseases can negatively affect breast milk composition include the systemic dissemination of inflammatory cytokines like IL-1, IL-6, PGE2, and TNF-β in the blood circulation that can be up-regulated by bacterial by-products. Based on results from this review, it can be hypothesized that milk from mothers with periodontal diseases might have low fat content, high levels of inflammatory mediators and fatty acids, changes in levels of immunoglobulins, and higher risk of bacterial dysbiosis, compared to milk from mothers with good periodontal health. (Prepared using Biorender.com).

Three main biological pathways might explain the relationship between the severity of periodontal disease and increased risks of adverse pregnancy outcomes. The first is inflammatory product circulation (shown in Figure 2) where the biosynthesis of pro-inflammatory cytokines in the blood serum and gingival fluid (such as IL-1b, IL-6, PGE_2_, and TNF-a) can be up-regulated by cellular exposure to bacterial products (Gibbs *et al*., 1992), such as the lipopolysaccharides of the gram-negative anaerobic bacteria of periodontal disease (e.g., *Tannerella forsythia, Porphyromonas gingivalis*). It is hypothesized that these endotoxins and pro-inflammatory cytokines could enter the bloodstream and reach the maternal–fetal interface (placenta and fetal membranes), thereby stimulating the production of PGE_2_ and TNF-alpha by the placenta (Gibbs *et al*., 1992, Romero *et al*., 1988), resulting in uterine contractility and early rupture of fetal membranes, resulting in preterm delivery (Gibbs *et al*., 1992).

This hypothesized mechanism has been supported by studies reporting high levels of PGE_2_, IL-1b, and IL-6 in the crevicular and amniotic fluid of birthing parents of preterm infants (Offenbacher *et al*., 1998, Dörtbudak *et al*., 2005). Another possible pathway that describes the relationship between periodontal diseases and adverse pregnancy outcomes is through the circulation of periodontal pathogens where they enter the bloodstream and invade the placenta (Han, 2011, Han *et al*., 2009). Intrauterine infections have been associated with placental colonization of *Fusobacterium nucleatum* and *Porphyromonas gingivalis* (Fardini *et al*., 2010, Dörtbudak *et al*., 2005). Inside the uterus, these pathogens might increase the synthesis of pro-inflammatory cytokines, prostaglandins, and metalloproteases derived from activated neutrophils (Dörtbudak *et al*., 2005), effects that could potentially stimulate uterine contraction and a preterm birth process (Dörtbudak *et al*., 2005). The risk of preterm birth could also be related to the immunological response against oral pathogens caused by periodontal disease (Han *et al*., 2009). Blood samples from the umbilical cords of preterm infants showed elevated levels of specific IgM directed against oral pathogens (Baskaradoss *et al*., 2012). This fetal immune reaction could be associated with an inflammatory response and/or both mechanisms could act in synergy to elevate the risk (Han *et al*., 2009).

#### Periodontal disease can affect human milk composition

A recent prospective cohort study (Badewy *et al*., 2022) was conducted by our research team to explore the impact of maternal oral inflammation on human milk composition, and how the oral inflammatory load (based on oral polymorphonuclear neutrophil counts) in birthing parents during the first 4 months postpartum impacts the immunological components and nutritional value of human milk including the human milk neutrophil counts and their activation state (based on the expression levels of cluster of differentiation [CD] biomarkers), as well as the content of lipids and fatty acids in human milk. The research was conducted to contribute to filling the knowledge gap in understanding the relationship between periodontal disease and human milk composition. Results from this study showed that birthing parents with moderate to severe oral inflammatory load exhibited a statistically significant decrease in CD64 biomarker expression, an increase in CD14 biomarker expression on human milk neutrophils, and a decrease in eicosapentaenoic acid (C20:5n-3) levels in their human milk at follow-up compared to baseline. This study reveals, for the first time, that maternal oral inflammation can influence human milk composition, emphasizing the need to consider how these alterations may impact long-term infant health outcomes.

#### Infants’ intestinal microbiome and maternal health

With human milk representing the protective agent against many infantile infections and long-term diseases, the potential impact of maternal oral inflammatory diseases on infant health is worth further investigation. The microbial colonization of the infant’s gut (i.e., the gut microbiome) acts as a barrier against various pathogens and is also essential for modulating the host’s metabolism as well as immune system development and maturation (Tanaka and Nakayama, 2017). Various factors influence the composition and development of the intestinal microbiota of the infant, over which the mother may have the greatest influence on the early acquisition and development of the infant’s microbiome (Richardson *et al*., 2018). The maternal microbial reservoir at various body sites plays a key role in the development of the infant’s microbiome due to the intimate relation birthing parents have with their infants during birth and while breastfeeding (Funkhouser and Bordenstein, 2013). The maternal microbiome can be transferred to the infants through five sources including fecal matter, vaginal fluids, and skin contact at the time of birth, and through human milk and skin contact after birth, particularly during breastfeeding, and oral bacteria transfer via the bloodstream (Funkhouser and Bordenstein, 2013). The development and composition of the infant’s gut microbiome thus depend on a large extent on maternal health (Richardson *et al*., 2018). For example, significant reductions in the abundance of *Bifidobacteria*, considered to be beneficial to health, have been reported in the human milk of allergic birthing parents and birthing parents with CD, compared to their healthy counterparts. (Grönlund *et al*., 2007) (Olivares *et al*., 2015).

**Figure 3.**
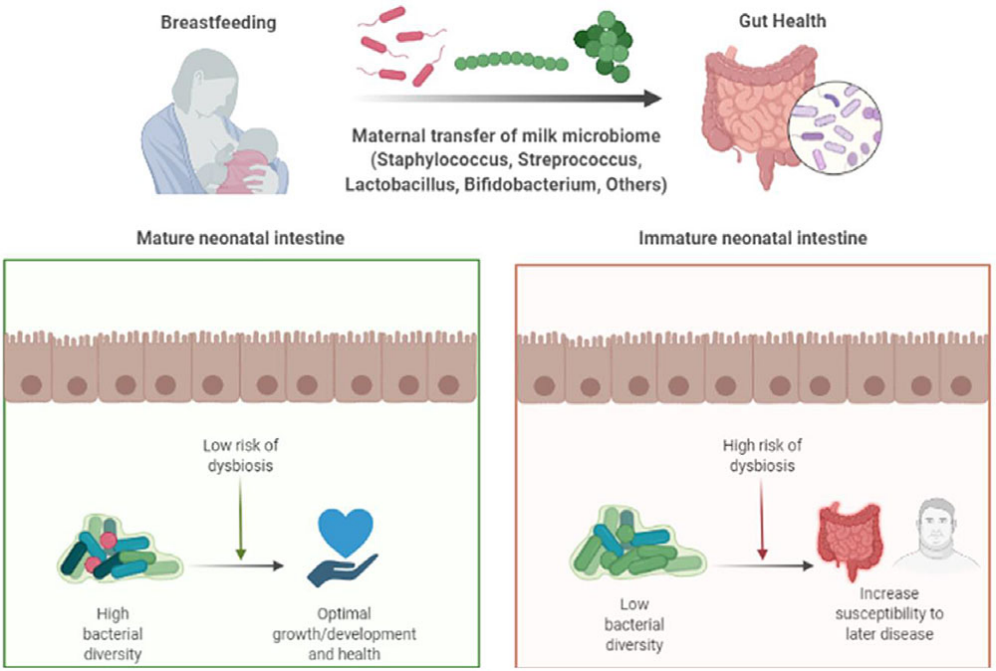
A simplified diagram showing the modulation of infant gut microbiota via breast milk microbiome. Infants receive various bacterial communities from breast milk which plays a major role in the development and maturation of the infants’ gut microbiome. Breast milk microbiome shapes the neonate’s intestinal maturation. Infants with mature intestine will have high bacterial diversity in their gut (i.e., low risk of bacterial dysbiosis) which protects them from later infections/diseases and is essential for the infants’ growth/development and optimal health. On the other hand, infants with immature intestine, will have low bacterial diversity in their gut (i.e., high risk of bacterial dysbiosis) which increases the infants’ susceptibility to later disease. (Prepared using Biorender.com).

The composition of an infant’s gut microbiome can have long-term implications on health and the development of its immune and nervous system, relationships which may be modulated by human milk (Figure 3). Various studies show that disturbances in the gut microbiome increase the infant’s susceptibility to autoimmune diseases such as diabetes, inflammatory bowel disease, atopy, and other conditions such as obesity (Arrieta *et al*., 2015, Turnbaugh *et al*., 2009, Fujimura *et al*., 2016). For example, evidence suggests that decreased abundance of *Bifidobacteria* in the infant gut is associated with obesity later in life (Abrahamsson *et al*., 2014). Additionally, low gut bacterial diversity (which may indicate a less mature microbiome) in the first month of life has been associated with asthma in children at the age of 7 years (Abrahamsson *et al*., 2014). Studies have also revealed associations between the gut microbiome and neurobehavioral outcomes in 7-year-old children including autism spectrum disorders and attention deficit hyperactivity disorder, suggesting that gut microbiome development can impact early brain development, resulting in behavioral changes in later life (Curran *et al*., 2016, Curran *et al*., 2015). With respect to cognitive development, a recent study (Carlson *et al*., 2018) investigated the association between gut microbiome in infancy and cognitive development at one and two years of age, where the development of the gut microbiome at one year was shown to predict cognitive communicative behaviors at two years of age. However, more robust longitudinal studies need to be conducted in order to assess the causal association between the studied variables.

### Research, practice, and policy implications

This review summarized existing evidence regarding the mechanisms related to periodontal diseases and its possible association with changes in human milk composition. There is an existing gap of knowledge regarding whether periodontal diseases in birthing parents can impact negatively the composition of their human milk. Future research in this topic could bring exciting outputs that will be significant not just to dentistry, but to obstetrics and gynecology, neonatology, and pediatrics to name a few.

#### Policy and practice implications

- Promoting oral health before and during pregnancy and in the postpartum period is important, but often neglected. Sixty-eight percent of obstetricians (Al-Habashneh *et al*., 2008) rarely or never encourage pregnant patients to seek dental treatment throughout their pregnancy.
- Training primary healthcare professionals in all capacities on the importance of educating birthing parents about the consequences of poor oral health may make the healthcare professionals more confident in addressing this issue with their patients (Heilbrunn-Lang *et al*., 2015).
- Studies showed significant increase in the confidence of midwives who received an oral health education training program, in promoting oral health among pregnant patients (Heilbrunn-Lang *et al*., 2015).
- If it appears that maternal periodontal inflammation also mediates negative effects on human milk, it will be critically important to make these facts known to the birthing parents’ healthcare providers and of course to birthing parents of newborns.
- This can be done by advocating for the incorporation of prenatal and postnatal oral health components within the curriculum of pediatric, obstetrics, and gynecology residencies training. With periodontal diseases affecting 20–50% of pregnant persons (Xiong *et al*., 2007, Madianos *et al*., 2001), the absence of dental care services can have negative impact on their oral health, quality of life, and health of their pregnancy and baby.
- Publicly funded dental programs for birthing parents who are affected by oral inflammatory conditions throughout pregnancy and postpartum do not exist in many countries (Northridge *et al*., 2020).
- Primary healthcare professionals need to be trained to do primary oral screening and referrals to dentists as required.

#### Research implications

- Findings from future studies that aim to investigate the impact of suboptimal oral health of birthing parents and human milk composition might aid in informing the development of a framework for policies and preventive programs to improve the oral health and access to dental care services for individuals of childbearing years.
- Future research in this topic could stress the importance of having public dental clinics in primary healthcare settings accessible to all pregnant and nursing individuals, where dentists can deliver basic restorative and preventive measures for birthing parents at-risk of periodontal diseases and suboptimal oral health, in particular individuals with low socio-economic status.

## Conclusion

Current evidence suggests that maternal infections and/or the presence of inflammatory disease adversely affect the composition and quality of human milk. Further, disturbances in the composition of an infant’s gut microbiome, in part due to alterations in human milk composition, is associated with adverse health trajectories for that infant and child long term. Given that periodontal diseases affect many individuals during pregnancy and this condition can progress *postpartum* if left untreated, optimizing and maintaining good oral health are critical for both the health of the mother and her baby. The biological mechanisms that may drive the relationships between periodontal diseases and poor pregnancy and infant outcomes include but are not limited to the systemic dissemination of inflammatory cytokines like IL-1, IL-6, PGE2, and TNF-β that can be up-regulated by bacterial by-products. There is every reason to hypothesize that periodontitis, just as mastitis, diabetes, and maternal infections could affect the composition of human milk, meaning that periodontitis could have similar effects on human milk inflammatory cytokines as well as lipids. Future efforts directed at investigating the potential association between periodontal diseases in individuals of childbearing years and the composition and quality of human milk will help to close the gap in our understanding, prevention, and treatment of oral diseases affecting pregnant and nursing individuals and advocate for integrating oral healthcare within primary healthcare settings.
